# The first 1000 days of the autistic brain: a systematic review of diffusion imaging studies

**DOI:** 10.3389/fnhum.2015.00159

**Published:** 2015-03-26

**Authors:** Eugenia Conti, Sara Calderoni, Viviana Marchi, Filippo Muratori, Giovanni Cioni, Andrea Guzzetta

**Affiliations:** ^1^Department of Developmental Neuroscience, Stella Maris Scientific Institute, IRCCS Stella Maris FoundationPisa, Italy; ^2^Department of Clinical and Experimental Medicine, University of PisaPisa, Italy

**Keywords:** autism, diffusion MRI, structural connectivity, toddlers, hyperconnectivity, neurodevelopment

## Abstract

There is overwhelming evidence that autism spectrum disorder (ASD) is related to altered brain connectivity. While these alterations are starting to be well characterized in subjects where the clinical picture is fully expressed, less is known on their earlier developmental course. In the present study we systematically reviewed current knowledge on structural connectivity in ASD infants and toddlers. We searched PubMed and Medline databases for all English language papers, published from year 2000, exploring structural connectivity in populations of infants and toddlers whose mean age was below 30 months. Of the 264 papers extracted, four were found to be eligible and were reviewed. Three of the four selected studies reported higher fractional anisotropy values in subjects with ASD compared to controls within commissural fibers, projections fibers, and association fibers, suggesting brain hyper-connectivity in the earliest phases of the disorder. Similar conclusions emerged from the other diffusion parameters assessed. These findings are reversed to what is generally found in studies exploring older patient groups and suggest a developmental course characterized by a shift toward hypo-connectivity starting at a time between two and four years of age.

## Introduction

Autism spectrum disorders (ASD) are a heterogeneous group of neurodevelopmental diseases affecting 1 in 68 children in the USA (Centers for Disease Control and Prevention [CDC], 2014), characterized by impairment in socio-communicative abilities, as well as restricted and stereotyped behaviors (American Psychiatric Association, 2013). The age at which clinical diagnosis is generally made ranges between 3 and 4 years, although converging evidence from both retrospective and prospective studies suggests that symptoms of ASD usually emerge in the first 2 years of life (Zwaigenbaum et al., 2013; Chawarska et al., 2014). This might suggest the diagnosis of ASD to be difficult within the first 1000 days, even if the symptoms emerge earlier, supporting the need for reliable early biological markers of the disorder.

An overwhelming number of studies have been performed in the last decade addressing the possible brain abnormalities associated with ASD. Increasing evidence suggests that in the phase when symptoms of ASD are fully expressed, i.e., in school-aged children or older, their neurobiological underpinnings consist of a disruption in structural and functional connectivity (reviewed in Vissers et al., 2012). Although the results are highly heterogeneous, both in terms of techniques used and clinical characteristics of the samples, a strong consensus now exists on the presence of an overall hypo-connectivity of long-range connecting tracts as a robust biological hallmark of the disease (Just et al., 2012; Aoki et al., 2013).

While the neurobiological underpinnings of ASD at a time when the clinical picture is well expressed are starting to be understood, much less is known on the preceding developmental course. This certainly reflects the course of symptoms, as the diagnosis is usually not considered stable before 30 months and infants diagnosed as being in the spectrum earlier are not rarely found to have grown out of the diagnosis later on (Turner et al., 2006). Precocious neuroanatomical correlates of ASD have been firstly detected by structural MRI studies reporting an increased brain volume (Sparks et al., 2002; Hazlett et al., 2005; Courchesne et al., 2011), stemming from both gray and white matter increase (Xiao et al., 2014), particularly in frontal and temporal regions (Schumann et al., 2010; Calderoni et al., 2012), or from an elevated extra-axial (extra-parenchymal) accumulation of cerebrospinal fluid (Shen et al., 2013). Furthermore, early anomalies of cortical development in key brain areas of ASD patients were also suggested by altered cortical thickness in superior/inferior frontal gyrus and superior temporal sulcus (Raznahan et al., 2013), or within central, intraparietal, and frontal medial sulci through sulcal shape analyses (Auzias et al., 2014).

Morphological anomalies described in ASD infants have been proposed as a correlate of the atypical organization of brain connectivity (Zhang and Sejnowski, 2000; Lewis and Elman, 2008). Indeed, the first years of postnatal life represent a crucial time period of brain development characterized in typical development by both axonal pruning and synaptogenesis to build up and strengthen cortical networks. Prospective studies in high-risk infants, recruiting newborns siblings of ASD children, have been recently set-up in several centers, often encompassing neuro-structural and neuro-functional measures of brain development and supporting the concept of ASD as a disorder of connectivity emerging during the first months of life (Bosl et al., 2011; Wolff et al., 2012).

A fuller understanding of the neurobiological transformations of the first 30 months of life, i.e., the first 1000 days, in children who will develop ASD is of utmost importance in order to support early clinical diagnosis and to allow for a prompt start of early behavioral interventions. In this paper, we aim to review current knowledge on the anomalies of brain structural connectivity in subjects with ASD in the first 30 months of life, age before which the diagnosis is still considered unstable (Landa, 2008). We in particular collected all studies focused on infants and toddlers with ASD exploring diffusion MRI and/or tractography.

## Methods

To review the literature, we queried PubMed and Medline using the following search terms: (ASD OR Autism OR ‘ASD^∗^’ OR Asperger) AND (Infant^∗^ OR Toddler^∗^) AND (‘Structural connectivity’ OR MRI OR DTI OR ‘diffusion imaging’ OR tractography). Criteria for inclusion in the study were established prior to the literature search. Inclusion was limited to papers published between January, 2000 and February, 2015, and was restricted to peer-reviewed English language articles. Reviews were not included in the selection, but were used to collect original studies. We screened all the selected papers in order to include studies in which mean age of the ASD sample was below 30 months. Studies focussing only on ASD subjects with known etiology (e.g., Fragile-X syndrome, tuberous sclerosis) were not included.

After duplicates removal, the search strategy yielded a total of 264 records. All abstracts were independently reviewed by three of the authors (EC, SC, and VM) and conflicting judgements were solved by consensus. During the review process 223 papers were excluded based on clear failure to meet the inclusion criteria. Forty-one papers were evaluated in full-text, of which 18 were review papers. Of the remaining 23, only four met the inclusion criteria and were included in this review (Ben Bashat et al., 2007; Wolff et al., 2012; Lewis et al., 2014; Xiao et al., 2014). All four were case-control studies, and only one of them was longitudinal (Wolff et al., 2012). Information on sample demographics, MRI characteristics and study design are reported in **Table 1**.

**Table 1:** Study characteristics.

Study (Design)	Groups (Mean in months ± SD)	Methods	Diffusion indexes	FA in ASD (*p* < 0.05)	↓FA in ASD (*p* < 0.05)	Other diffusion indexes *p* < 0.05
Ben Bashat et al. (2007) (Case control)	ASD: *n* = 7 (29 ± 7) TD: *n* = 41 (38 ± 13)	DTI Whole-brain ROI analysis	FA Probability Displacement	CC (genu and splenium); left PLIC; left external capsule; left forceps minor; left CST	n/a	**Probability in ASD:** bilateral forceps minor; left external capsule; left PLIC; blateral ALIC; bilateral CST ↓ **Displacement in ASD:** blateral forceps minor; left external capsule; left PLIC; blateral ALIC; left CST
Wolff et al. (2012) (Longitudinal)	**6 months** HR+: *n* = 28 (6.8 ± 0.8) HR–: *n* = 64 (6.7 ± 0.8) **12 months** HR+: *n* = 17 (12.7 ± 0.7) HR–: *n* = 49 (12.7 ± 0.6) **24 months** HR+: *n* = 17 (24.5 ± 0.6) HR–: *n* = 33 (24.7 ± 0.8)	DTI ROI analysis	FA AD RD	**FA in HR+ (6 months):** CC (body); left Fornix; left ILF; bilateral PLIC; left UF	↓**FA in HR+ (24 months):** left ALIC; left anterior thalamic radiation	No significant differences in AD and RD
Xiao et al. (2014) (Case control)	ASD: *n* = 50 (29.9 ± 5.54) DD: *n* = 28 (28.2 ± 4.4)	DTI VB analysis	FA MD	**FA in ASD:** CC; posterior cingulate; limbic lobe	n/a	↓ **MD in ASD:** CC; posterior cingulate; left limbic lobe; left Insula
Lewis et al. (2014) (Case control)	HR+: *n* = 31 (24.5 ± 0.7) HR–: *n* = 82 (24.7 ± 0.9) LR: *n* = 23 (24.5 ± 0.6)	DTI Whole-brain Network analysis	Local and Global Efficiency			**Local efficiency in ASD:** bilateral T and O lobes (inferior and medial regions), and predominately in the RH for lateral regions, extending to the supramarginal gyrus; the left T and O lobes, extending into the precuneus, and in several posterior regions in the RH ↓ **Global efficiency in ASD:** bilateral T and O lobes, extending in the RH to the angular and supramarginal gyri. The left pars triangularis and medial orbital gyrus; bilateral T lobes, extending in the left hemisphere to the O cortex, and in the RH to the angular and supramarginal gyri

*AD, axial diffusivity; ALIC, anterior limb of internal capsule; CC, corpus callosum; CST, cortico-spinal tract; DD, developmental delay; FA, fractional anisotropy; HR-, high-risk subjects, negative; HR+, high risk subjects, positive; ILF, inferior longitudinal fasciculus; LR, low risk subjects; MD, mean diffusivity; O, occipital; PLIC, posterior limb of internal capsule; RD, radial diffusivity; RH, right hemisphere; T, temporal; TD, typical developing group; UF, uncinate fasciculus.*

Mean age of the ASD group across the studies varied between 6 and 29 months. In particular Wolff et al. (2012) assessed a longitudinal cohort of high-risk subjects (ASD siblings) at 6, 12, and 24 months. Subjects were grouped retrospectively, according to the diagnosis at 2 years, into high-risk positive (HR+; siblings who obtained scores over ASD cut off at ADOS (Lord et al., 2000) evaluation) and high-risk negative (HR–; siblings who obtained scores below ASD cut off at ADOS evaluation; **Table 1**). In the remaining three studies (Ben Bashat et al., 2007; Lewis et al., 2014; Xiao et al., 2014), the ASD populations were more homogeneous with mean age ranging from 24 to 29 months. Lewis et al. (2014) analyzed a population of ASD siblings with small age range (24.5 months ± 0.73). They compared HR+ and HR- with subjects with typical development. Xiao et al. (2014) compared ASD subjects (29.92 months ± 5.54) with age-matched toddlers with developmental delay (DD). Lastly, Ben Bashat et al. (2007) analyzed ASD and typically developing subjects; while the ASD sample was entirely composed of toddlers (29 months ± 7), typical subjects encompassed a wider age range, i.e., 4 months–23 years.

MRI methodology used in the studies was heterogeneous. Ben Bashat et al. (2007) were the only to use a 1.5T MRI scanner with diffusion at six directions and a *b*-value of 6000. The remaining three studies utilized a 3T MRI scanner, and diffusion acquisition was based on either 25 (Wolff et al., 2012; Lewis et al., 2014) or 30 directions (Xiao et al., 2014) with a *b*-value of 1000. Post-processing analyses were similarly, heterogeneous. Lewis et al. (2014) used a novel approach based on the assessment of network efficiency. By using diffusion data the authors obtained measures of the length and strength of connections between anatomical regions and thus calculated efficiency of sets of connections (networks), rather than considering each connection independently, obtaining measures of local and global nodal efficiency. The remaining three papers used more conventional approaches FA, MD, and probability and displacement diffusion indexes, with two studies using a ROI-based analysis (Ben Bashat et al., 2007; Wolff et al., 2012) and one using a voxel-based analysis (Xiao et al., 2014).

## Results

Findings are reported as grouped in (i) FA, which is currently the most common index explored in diffusion studies, and (ii) all other diffusion indexes.

### Fractional Anisotropy

**FIGURE 1:**
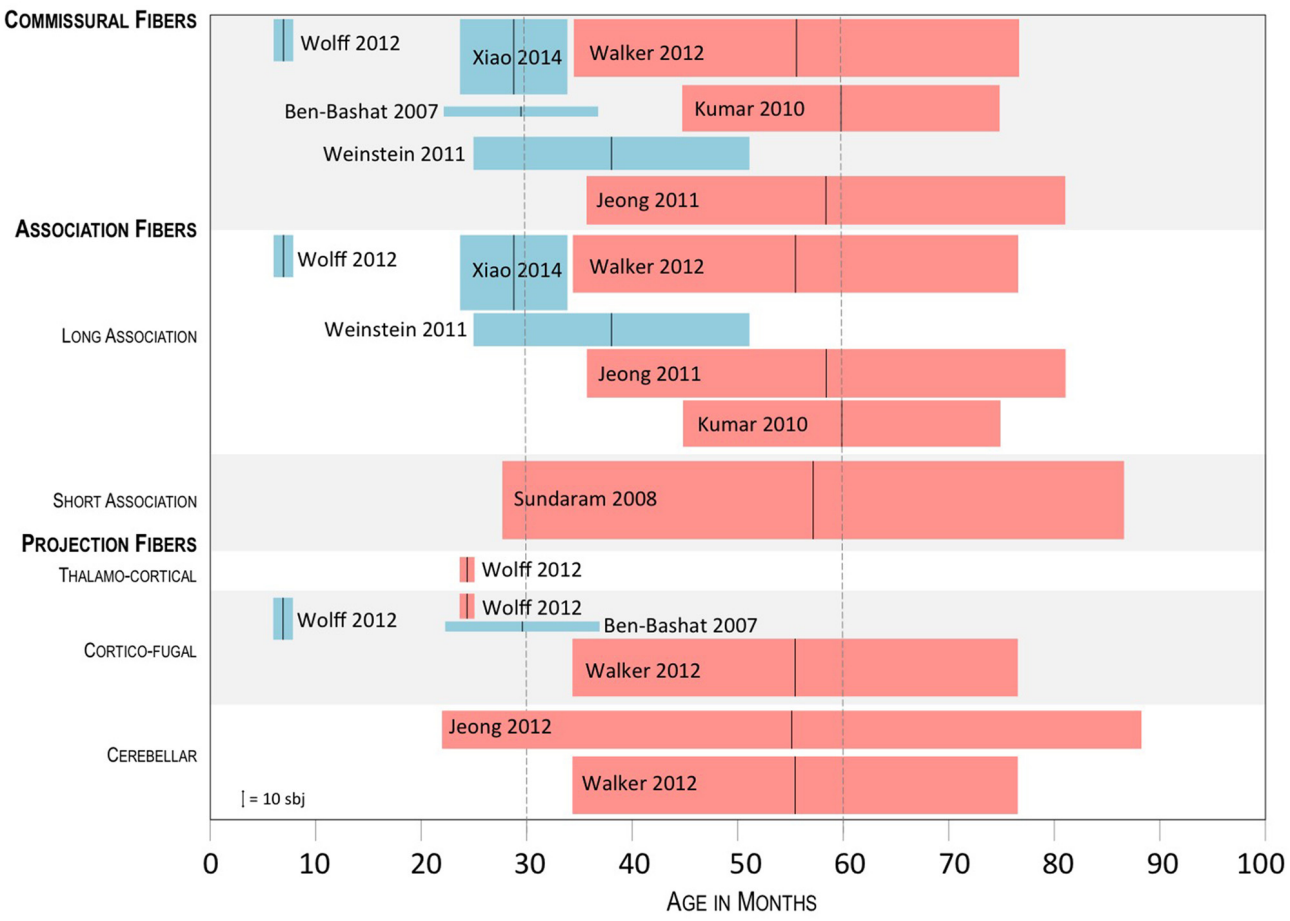
**Summary of the results of studies exploring FA in ASD subject groups whose mean age was below 60 months.** Tracts showing a significant difference between ASD and controls are shown, with blue boxes indicating higher FA in ASD and red boxes indicating lower FA in ASD. The black vertical bar in the middle of the boxplots shows the mean age of the ASD group; the lateral sides of the boxplots, show the SD; height of the boxplots represents the sample size, as referred to in the lower left corner.

Three of the four studies measured FA values. Findings are discussed according to the identified connections as grouped into commissural fibers, projection fibers, (thalamocortical, corticofugal, and cerebellar), and association fibers (long and short range) (Catani et al., 2012). As FA is widely explored in most diffusion studies, in order to put our results into a developmental perspective, we compared the three selected studies with those exploring structural connectivity in older subjects, and in particular in ASD groups were the mean age was between 30 and 60 months (**Figure 1**).

*Commissural fibers (corpus callosum, anterior commissure, posterior commissure, forceps major, and minor)*. In three (Ben Bashat et al., 2007; Wolff et al., 2012; Xiao et al., 2014) of the four selected papers commissural fibers were explored. In all cases, significantly higher FA values were found in infants and toddlers with ASD as opposed to control subjects. In particular, Xiao et al. (2014) showed higher FA values in the splenium of the corpus callosum, while Ben Bashat et al. (2007) also in the genu, and in the left forceps minor. Wolff et al. (2012) reported significant differences within the body of corpus callosum at 6 months of age, which were not observed at 12 and 24 months. The comparison with studies exploring older subjects (mean age 30–60 months), showed similar findings in an ASD group with mean age of around 38 months, with higher FA values in the ASD group in the genu and the body of the corpus callosum (Weinstein et al., 2011), and reverse results in three further studies exploring groups of subjects with mean age between 56 and 60 months, with lower FA values in the ASD group in all parts of the corpus callosum (Kumar et al., 2010; Jeong et al., 2011; Walker et al., 2012).

*Association fibers (long: arcuate fasciculus, cingulum, uncinate fasciculus, inferior fronto-occipital fasciculus, fornix; short: U-shape fibers).* In two of the four selected papers association fibers were explored (Wolff et al., 2012; Xiao et al., 2014). Again, in both cases, significantly higher FA values were found in infants and toddlers with ASD as opposed to control subjects. In particular, Xiao et al. (2014) found higher FA values in the cingulum and the limbic lobe, while Wolff et al. (2012), found higher FA values in the left fornix and uncinate fasciculus at 6 months, which were not observed later, at 12 and 24 months. The comparison with studies exploring older subjects, showed higher FA values in ASD subjects with mean age of 38 months (Weinstein et al., 2011), in particular within the left superior longitudinal and arcuate fasciculus. In other four studies, focussed on children ranging from 56 to 60 months, lower FA values were reported in the superior temporal gyrus (Walker et al., 2012), uncinate fasciculus and arcuate fasciculus (Kumar et al., 2010; Jeong et al., 2011), inferior-frontal-occipital fasciculus, superior longitudinal fasciculus, and cingulum (Kumar et al., 2010), and short intra-lobar frontal fibers (Sundaram et al., 2008).

*Projection fibers (internal capsule; external capsule; cerebellar tracts; corona radiata corticofugal fibers, thalamocortical fibers).* In two of the four selected papers projection fibers were explored (Ben Bashat et al., 2007; Wolff et al., 2012). In both cases, significantly higher FA values were found in infants and toddlers with ASD as opposed to control subjects, except for Wolff et al. (2012) who reported increased FA values at 6 months of age within the right posterior limb of internal capsule and interestingly decreased FA values within the left anterior thalamic radiation at 24 months. Ben Bashat et al. (2007) reported increased FA values in left posterior limb of internal capsule, in left external capsule and in left cortico-spinal tract. Studies exploring older subjects found lower FA values in ASD subjects in cortico-spinal tract, pons, and posterior limb of internal capsule (Walker et al., 2012). Significant differences in the cerebellar tracts were only found in two studies assessing children with mean age between 30 and 60 months. Interestingly, they both found lower FA values in ASD subjects within the cerebellum (Walker et al., 2012) and the dento-rubrothalamic projection (Jeong et al., 2012).

### Other Diffusion Indexes

Three of the four papers explored diffusion indexes different from FA. The most innovative approach was the one by Lewis et al. (2014) who explored network efficiency between nodes, either local or global. They found decreased local and global efficiency over temporal, parietal, and occipital lobes in high-risk infants later classified as ASD, as compared to both low- and high-risk infants not classified as ASD. The frontal lobes showed only a reduction in global efficiency in Broca’s area. The reductions in nodal local and nodal global efficiency in ASD infants were interpreted not simply as indexes of hypo-connectivity, but rather as the result of more limited local connectivity and less direct connections to other brain regions. Ben Bashat et al. (2007) calculated probability and displacement values using q-space analysis for the diffusion data set, according to their own previous papers, claiming them as better indicators of white matter pathology than conventional FA (Assaf et al., 2002; Ben Bashat et al., 2005). Hyper-connectivity, reflected in significantly increased probability along with reduced displacement values, was detected in several brain regions. In particular, significant increase in probability values (and decrease in displacement values) were reported in the left forceps minor, left external capsule, left posterior limb of the internal capsule and bilaterally in the anterior limb of the internal capsule and cortico-spinal tract. Finally, Xiao et al. (2014) calculated MD values in ASD toddlers showing a reduction in the same regions where FA was increased, including the corpus callosum, cingulum, and limbic lobe. Overall, these results support evidence of hyper-connectivity in infants and toddlers with autism and are in contrast with the decreased, restricted diffusion reported in previous studies in older children and adolescents.

## Discussion

Evidence from the reviewed studies suggests that white matter atypical diffusion properties are a consistent finding in the early phases of ASD, and can precede the full-blown expression of the clinical picture. All three studies exploring brain FA, the most commonly investigated diffusion measure (Basser and Pierpaoli, 1996), extensively reported higher values in ASD subjects compared to controls. Along the same lines, the majority of the other diffusion measures explored, including MD, probability, and displacement values, points toward an increased directional restriction of water diffusion in several brain tracts. Interestingly, these findings are opposite to what reported in an overwhelming body of literature showing a widespread reduction of FA and increase in MD in children, adolescents and young adults with ASD (Aoki et al., 2013).

While these latter reports have been considered by most as supporting the underconnectivity theory of ASD, firstly formulated from functional MRI studies (Just et al., 2004), recent studies have underlined the importance of not over-interpreting diffusion data as direct indices of structural connectivity, particularly in consideration of the methodological limits expressed by diffusion tensor imaging (Jones et al., 2013; Castellanos et al., 2014). Indeed, the significance of diffusion indices as to brain connectivity is increasingly matter of debate, and it is strongly encouraged that more advanced approaches, such as the HARDI (high angular resolution diffusion imaging), are applied in order to minimize the limitations of DTI, including a better resolution of crossing and kissing fibers (Bloy et al., 2012).

The relationship between structural and functional connectivity is not fully understood in subjects with ASD, and particularly so in infants and toddlers, since no study to date has specifically addressed this issue (Uddin et al., 2013). Initially, based on fMRI studies, local regions of hyper-connectivity described in high functioning ASD subjects were interpreted as related to islands of preserved capacities (Wass, 2011). More recently, however, functional hyper-connectivity has been interpreted as reflecting diffuse and less specialized, rather than more efficient, brain networks in older subjects (Just et al., 2012).

The few functional connectivity studies performed in infants and toddlers with ASD do not clarify this issue. In the only EEG study assessing connectivity in infants with ASD, Orekhova et al. (2014) demonstrated elevated alpha range connectivity, particularly in frontal and central areas, by applying High Density-EEG in high-risk infants aged around 14 months (range 12–17), who were later diagnosed with ASD. Conversely, in older ASD patients aged around 29 months (range 12–46), Dinstein et al. (2011) reported significant weaker functional connectivity in putative language areas, as assessed by sleep fMRI inter-hemispheric synchronization.

Irrespective of its connotations, the issue remains about the time of development at which, as compared to control subjects, white matter properties begin to shift from increased FA to decreased FA. Overall, studies in this review suggest a time between 2 and 4 years of age as the phase when this shift is most likely starting to occur. The first structures showing an inversion of FA distribution are the thalamocortical projections, which were found to have significantly lower FA values in ASD toddlers already at 24 months of age (Wolff et al., 2012). Around the same age, a decreased local and global efficiency over temporal, parietal and occipital lobes in ASD toddlers was found and interpreted as the result of connectivity disruption (Lewis et al., 2014). A more widespread shift of FA, involving in particular commissural and long-association fibers, is likely to occur at a slightly older age, as shown by the global persistence of increased FA in study cohorts with mean age up to 38 months (Ben Bashat et al., 2007; Weinstein et al., 2011; Xiao et al., 2014). In most papers assessing populations of older children, starting from those with mean age of around 5 years, higher FA is generally not found, with few exceptions. One of them is the report by Billeci et al. (2012) who found a widespread FA increase in the white matter of ASD young children aged around 5.5 years, particularly at the level of corpus callosum, cingulum, internal capsule, and arcuate fasciculus. In their study, however, the regression analysis of FA vs. age showed that FA increase was mainly attributable to the subgroup of younger patients, with the older ones indeed showing an FA reduction.

While interpreting the findings of the papers included in this review, the heterogeneity in the quality of the studies needs to be mentioned. For instance, number of diffusion directions used ranged from 6 in one study to 25 or 30 in the other three. Also, movement artifacts are likely to have affected differentially the findings, as only two of the four studies (Ben Bashat et al., 2007; Xiao et al., 2014) used sedation during scanning, which is known to significantly reduce motion artifacts as compared to natural sleep scanning. Given the low number of studies collected, however, it would be hard to properly ponder the contribution of the methodological differences among the studies as to the interpretation of the findings.

Overall, the results of this review describe a developmental trajectory of white matter diffusion properties in ASD infants compatible with the early signs of enlarged head circumference and macrocephaly extensively reported in the literature (Courchesne et al., 2011). Converging evidence from head-circumference, neuroimaging, and post-mortem studies supports the view that the brains of ASD individuals are larger than controls from at least 2 years of age, particularly at the level of the frontal and temporal lobes (Courchesne et al., 2011; Muratori et al., 2012). This finding tends to disappear within the preschool age and eventually results in significant undergrowth during adolescence. This trajectory greatly parallels the developmental course in diffusion indices emerging from this review and is compatible with the proposed interpretation of early overgrowth as an epiphenomenon of abnormal connectivity resulting from a lack of physiological synaptic pruning (Frith, 2003).

Despite the growing interest in the literature in understanding the neuro-structural underpinnings of ASD at the onset of clinical symptoms, or before, available data are still not exhaustive. To date, studies are still scarce and are highly heterogeneous in terms of sample size, control groups and MRI analysis. A possible strategy to face this limitation would be the partaking of large, longitudinal ASD data repositories within the scientific community, which would provide priceless information about the early neurostructural markers of the disorder. The increasing application of this and other methodological strategies, including the use of higher-order diffusion imaging such as HARDI, is likely to rapidly and exponentially widen in the near future our knowledge on the developmental trajectory of early brain connectivity in ASD.

## Conflict of Interest Statement

The authors declare that the research was conducted in the absence of any commercial or financial relationships that could be construed as a potential conflict of interest.

## ABBREVIATIONS

ASD: autism spectrum disorders
FA: fractional anisotropy
HD-EEG: high-density electroencephalography
HR: high risk
MD: mean diffusivity.
